# Resolving Steam Turbine Casing Thermal Management Challenges with a Dual Attentive Bi-GRU Soft Sensor for Transient Operation

**DOI:** 10.3390/ma18225213

**Published:** 2025-11-18

**Authors:** Sylwia Kruk-Gotzman, Grzegorz Bzymek, Konrad Kania

**Affiliations:** 1 Energy Conversion Department, Institute of Fluid-Flow Machinery Polish Academy of Sciences, 80-231 Gdańsk, Poland; 2 PGE Mining and Conventional Energy plc, Opole Power Plant, 45-920 Opole, Poland; grzegorz.bzymek@gkpge.pl; 3 Department of Quantitative Methods in Management, Faculty of Management, Lublin University of Technology, 20-618 Lublin, Poland; k.kania@pollub.pl

**Keywords:** soft sensor, Bi-GRU neural network, attention mechanism, turbine casing temperature prediction, operational regimes, thermal stress monitoring

## Abstract

This study introduces a novel dual-model deep learning framework based on Bidirectional Gated Recurrent Units (Bi-GRUs) with the Attention Mechanism to predict intermediate-pressure (IP) turbine casing temperatures in a 370 MW coal-fired power plant under varying operational regimes, including startup, shutdown, and load-following conditions. Accurate temperature prediction is critical, as thermal gradients induce significant stresses in the turbine casing, potentially causing fatigue crack initiation. To mitigate sensor failures, which lead to costly downtime in power generation systems, the proposed soft sensor leverages an extensive dataset collected over one year from Unit 4 of the Opole Power Plant. The dataset is partitioned into shutdown and active regimes to capture distinct thermal dynamics, enhancing model adaptability. The framework employs advanced preprocessing techniques and state detection heuristics to improve prediction robustness. Experimental results show that the dual-model approach outperforms traditional machine learning models (Random Forest Regressor, XGBoost) and single-model deep learning baselines (LSTM, Single Attentive Bi-GRU), achieving a mean squared error (MSE) of 2.97 °C and a mean absolute error (MAE) of 1.07 °C on the test set, while also maintaining low prediction latency suitable for real-time applications. This superior performance stems from a tailored architecture, optimized via Hyperband tuning and a strategic focus on distinct operational regimes. This work advances soft sensing in power systems and provides a practical, real-time solution for stress monitoring and control, particularly as coal plants in Poland face increased cycling demands due to the growth of renewable energy sources, rising from 7% in 2010 to 25% by 2025. The approach holds potential for broader application in industrial settings requiring robust temperature prediction under variable conditions.

## 1. Introduction

In power generation systems, physical sensors are critical for monitoring key operational parameters, such as temperature, pressure, and flow. However, these sensors are not immune to failure. When a sensor malfunctions—whether due to wear, thermal stress, or mechanical damage—its replacement or repair often requires shutting down the equipment, a process that can be both costly and logistically challenging. In the context of a coal-fired power plant, halting a 370 MW turbine, such as the 18K370 unit at the Opole Power Plant (Unit 4), is a significant undertaking, involving weeks of downtime, lost production, and complex restart procedures. This article explores a hypothetical yet plausible scenario: the failure of a sensor measuring the casing temperature of the turbine’s intermediate-pressure (IP) stage. Immediate physical intervention is impractical, yet accurate temperature data remains essential for safe and efficient operation. To address this challenge, we introduce a soft sensor—a computational model that estimates unmeasurable or missing process variables using available data from other sensors and historical records [1,2]. Unlike traditional hardware sensors, a soft sensor is a virtual tool, employing mathematical models or machine learning to provide real-time predictions without physical installation. In this case, it serves as a lifeline, delivering critical IP casing temperature estimates when a physical sensor fails.

This is particularly vital because casing temperature directly influences thermal gradients—differences between the steam and metal surfaces that drive stress and fatigue. Thermal stresses translate into mechanical stresses, which must not exceed [3] permissible limits to prevent material damage. During startups and shutdowns, thermal gradients can spike rapidly, risking thermal shock if not properly monitored and controlled. Typical variations in temperature and mechanical stress that the inner casing undergoes during each steam turbine cycle (startup to shutdown) include a rapid temperature rise and corresponding surge in thermal stresses during startup, a quasi-steady thermal and mechanical state during full-load operation, and a gradual cooling phase accompanied by stress reversal and decay as the turbine shuts down. During steady-state operation, conditions stabilize, but startup induces large temperature gradients leading to compressive stress, and shutdown leads to a drop in temperature, causing tensile stresses. Therefore, the steam turbine set is usually controlled and protected by an online stress control [4,5]. Delayed or absent temperature data could lead to unsafe ramping rates, exacerbating structural damage.

The need for such solutions has grown in Poland’s evolving energy landscape. Over the past 15 years, renewable energy has surged from 7% of the national mix in 2010 to Poland aiming for 32% (per its 2021 NECP, with upward revisions suggested) [6], driven by EU policies and carbon price increases—from €5 per ton in 2015 to a projected €90 per ton by the end of 2025 [7]. This shift has forced coal plants, traditionally designed for steady base-load operation, into flexible cycling modes, with annual startups rising from a handful to dozens. Table 1 presents the number of startups for the analyzed Unit 4 across two years, 2023 and 2024. The startups are categorized based on the thermal state of the unit prior to startup. The increase in total startups from 25 in 2023 to 39 in 2024 indicates a higher frequency of operational cycling. Some latest research work related to this topic can be found in [8,9].

### 1.1. Online Steam Turbine Stress Control

The 370 MW sub-critical unit at the Opole Power Plant [10], powered by the 18K370 steam turbine, converts thermal energy from coal combustion into electrical power via the Rankine cycle. The system comprises a BP-1150 boiler, the 18K370 condensing turbine with high-pressure (HP), intermediate-pressure (IP), and low-pressure (LP) stages, and a GTHW-370 generator, achieving an efficiency of approximately 36–38%. The turbine casing, forged from chromium–molybdenum steel, encloses these stages and endures significant thermal and mechanical stresses. Based on operational data from the Opole Power Plant, the steam turbine operates as follows: steam is generated in the boiler at 16.5–18 MPa (165–180 bar) and 535–540 °C, entering the HP stage of the 18K370 turbine. Here, it expands to 4–5 MPa (40–50 bar) and 350–400 °C. After reheat to 520–540 °C, it flows into the IP stage, dropping to 0.2–0.6 MPa (2–6 bar) and 300–350 °C. Finally, it exhausts from the LP stage at 5–7 kPa (0.05–0.07 bar) and 35–40 °C into the condenser.

**Figure 1:**
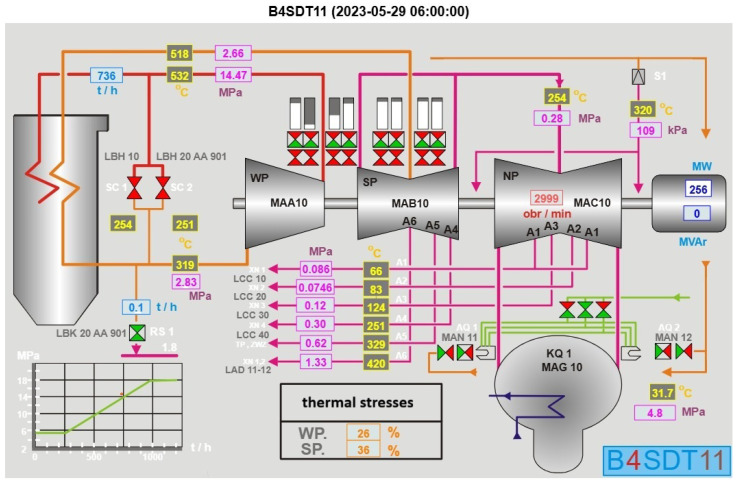
SCADA screen-view of the TG4 power plant unit, showing the flow of steam and locations where thermal stresses are monitored.

The schematic of the power plant (Figure 1) illustrates how thermal stresses are tracked across the turbine stages. The integrity of a turbine casing is highly dependent on temperature gradients. Rapid temperature fluctuations during transitional operations can induce significant thermal stresses on the components of the system. In particular, the thick-walled casing is exposed to the following challenges across various operational modes:

**Base-Load Operation:** At a steady output of 370 MW, temperature gradients stabilize (e.g., 50–80 °C across the casing thickness), minimizing thermal stresses.

**Figure 2:**
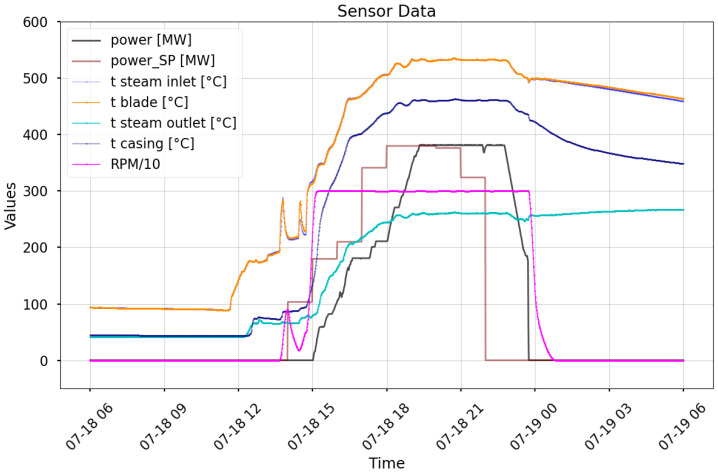
Automation system response showing temperature and power trends during a startup. Around 14:00, a significant mismatch—up to 200 °C—occurred between the steam temperature (blue, mostly hidden behind the orange blade line due to rapid thermal equalization) and the IP casing temperature (navy). Since the blades quickly reach steam temperature, their curve typically overlaps and conceals the steam line. In this case, the temperature difference triggered a corrective response: the steam was cooled and the turbine speed was reduced.

**Cycling and Startups:** Frequent startups and shutdowns introduce rapid but relatively small temperature changes, as the turbine does not fully cool down between cycles, resulting in a higher initial metal temperature and lower thermal gradients. This mitigates excessive thermal stress and reduces the risk of fatigue cracking. In contrast, infrequent startups following prolonged shutdowns induce both rapid and substantial temperature changes, as turbine components cool to ambient conditions and must be reheated to operational temperatures exceeding 400 °C. For instance, during a cold start, the initial steam–metal temperature mismatch can reach values on the order of 100 °C or more. The IP casing experiences a temperature increase of 400 °C within several hours (cf. Figure 2), risking thermal shock—uneven expansion that can cause micro-cracks or distortions. Table 2 presents the relationship between the secondary steam temperature and power output. The parameters can be directly traced from the raw signals in Figure 2.

**Reducing Load and Shutdowns:** Load reduction leads to a drop in steam temperature, generating non-uniform thermal gradients across the HP, IP, and LP casings. Due to its greater wall thickness, the high-pressure (HP) casing cools more slowly than the intermediate- and low-pressure sections, creating differential contraction that imposes stress on welds and joints. Additionally, differences in thermal inertia between the rotor and the interstage seals further intensify thermal stress. The rotor, having a much larger thermal mass, dissipates heat more slowly than the lightweight seals, leading to uneven contraction. This mismatch can cause the labyrinth strips to loosen or shift, jeopardizing both sealing effectiveness and mechanical integrity. To minimize such effects, cooling must proceed more gradually than heating, allowing for smoother temperature equalization within the structure. Continuous monitoring is therefore essential, as applying a large temperature differential to a partially cooled turbine may trigger severe damage.

The objective of steam turbine stress control is to raise the turbine rotor and casing temperatures to operational levels as quickly as possible without exceeding material limits and shortening component life [4,11]. In the analyzed power plant, the following allowable values of metal temperature gradients during heating (startup) and cooling (shutdown) have been established to ensure safe operation [10]:

- HP inner casing: −1.64 °C/min to +3.28 °C/min;
- IP inner casing: −1.35 °C/min to +2.70 °C/min.

The initial mismatch is controlled automatically through system permissives before allowing steam admission to the IP turbine. Temperature ramping can only start once the HP, IP, and LP turbine sections have been sufficiently heated. If allowable stress limits are exceeded, the automation system responds by adjusting operational parameters. For example, it may reduce the rate of steam temperature change, limit output power, or modify steam flow to minimize the risk of damage. Such proactive measures are crucial for preventing crack formation and ensuring the long-term reliability of the turbine casing.

Figure 2 shows the outcome of the automation system’s response to a thermal mismatch of up to 200 °C between the steam temperature (orange) and the IP casing temperature (navy). Exceeding material stress thresholds prompted the system to reduce steam temperature and turbine speed.

### 1.2. Soft Sensors

Soft sensors leverage existing sensor data and historical records to predict critical parameters, such as the IP casing temperature in this study, without requiring additional hardware.

Prior to the adoption of data-driven approaches, physics-based thermal models were commonly employed to estimate rotor or casing temperatures in steam turbines. In some cases, these models were augmented with estimation filters (e.g., Kalman filters) to infer internal thermal states from measurable signals such as bearing temperature, rotational speed, and live steam parameters (pressure and temperature) [4,12]. Examples of online thermal stress monitoring can be found in the use of equivalent Green’s functions and Duhamel’s integral [13], as well as in approaches incorporating variable heat transfer coefficients and temperature-dependent material properties under transient conditions [14]. However, these methods require accurately defined model parameters and involve considerable computational complexity, which limits their applicability for real-time use in embedded systems with constrained processing capabilities [15].

Over the past two decades, data-driven soft sensors have been extensively explored across a wide range of industrial applications. Early implementations demonstrated their effectiveness in chemical processes, where neural networks were used to infer unmeasurable variables such as concentration and temperature [1]. In the context of power systems, soft sensors have been applied to predict quantities such as power output, emissions, and temperature distributions. For instance, machine learning-based soft sensors—specifically Random Forest and ResNet models—were shown to outperform a physically based numerical model in forecasting photovoltaic panel temperature, including in challenging floating PV installations where environmental variability is high [16]. Another example is the use of an online ensemble bagging regression (OEBR) model for short-term wind power forecasting, where the target variable was the instantaneous active power output of wind farms [17]. Although predictive accuracy was only marginally lower than in previously reported data-driven methods, the approach offered a substantial reduction in computation time—an important advantage for real-time deployment.

More recently, deep learning techniques have gained prominence for their capacity to model complex, nonlinear dependencies in time series data. Recurrent Neural Networks (RNNs), particularly Long Short-Term Memory (LSTM) and Gated Recurrent Unit (GRU) models, have shown promising results in soft sensing tasks. A bidirectional LSTM (Bi-LSTM) model optimized with the adaptive wind-driven optimization (AWDO) algorithm was proposed to forecast load at both individual and aggregated levels [18]. The model achieved an average RMSE of 0.121 kW and MAPE of 7.55% for individual loads, and 0.025 kW and 1.51% for aggregate loads, demonstrating its ability to extract meaningful patterns from complex temporal data.

Unlike load-following operating conditions—where variables such as temperature, pressure, or rotational speed vary to a limited extent and are often well approximated by simplified physical models—startup and shutdown regimes exhibit strong nonlinearities and dependencies on initial conditions. Prediction becomes especially challenging at the onset of regime transitions—for example, when the system shifts from shutdown to startup—as such moments involve abrupt changes in behavior that are difficult to anticipate using historical patterns. To address these complexities, a data-driven classification strategy identifies steam turbine operating modes by predicting rotational speed (RPM) from transient sensor patterns [19]. Utilizing decision trees and k-nearest neighbors trained on high-frequency measurements, the models achieved over 93% accuracy in distinguishing steady-state from transient conditions, demonstrating robust generalization across turbine types for real-time condition monitoring.

While specific examples of deep learning models successfully handling transitional regimes in power systems remain scarce in the literature, related work from other domains demonstrates promising architectural solutions. Accordingly, to effectively address unsteady-state processes characterized by rapid transients and dynamic changes, advanced architectures combining bidirectional GRUs (Bi-GRUs), attention mechanisms (AM), and convolutional layers are employed. The incorporation of the AM in encoder-decoder LSTM models has led to improved predictive performance—for instance, in temperature forecasting of permanent magnet synchronous motors, where attention-enhanced models outperformed their baseline counterparts [20]. Similar enhancements have been demonstrated in industrial quality prediction using Temporal Convolutional Networks (TCN) with both spatial and temporal attention layers [21]. Hybrid architectures combining Bi-GRUs, multi-scale convolutional layers, and space- and time-wise attention have further shown their effectiveness in capturing spatial and temporal dependencies relevant to soft sensing tasks [22].

Building on this foundation, this study integrates a dual Bi-GRU architecture with the Attention Mechanism to develop a soft sensor for predicting turbine casing temperatures under varying operational regimes. To address the limitations of existing methods in capturing nonlinearities and operational variability, the proposed approach introduces a data partitioning strategy based on the operational state of the turbine—specifically, whether it is active or offline. To the best of the authors’ knowledge, this method has not been previously reported. In addition to improving predictive accuracy, the proposed framework offers scalability for real-time monitoring applications in turbine systems. We aim to find the simplest architecture that still delivers robust performance. This article outlines a set of noteworthy contributions, which can be summarized as follows:

- Development of a dual-model architecture ($m_{\mathrm{shutdown}}$ and $m_{\mathrm{active}}$) using Bi-GRUs with Attention Mechanism, tailored to handle the distinct dynamics of shutdown and active regimes, addressing thermal hysteresis in temperature prediction.
- Implementation of a data partitioning strategy based on operational regimes, ensuring data continuity for robust training and evaluation across diverse operating conditions.
- Optimization of the model using Hyperband tuning, achieving a simplified yet effective architecture with a *L* of 30 time steps, validated by a lowest MSE of 2.97 °C on the test set.
- Demonstration of superior performance over traditional machine learning and single-model deep learning approaches, with practical implications for real-time soft sensing in power generation systems.

The paper is structured as follows: Section 2 details data and preprocessing, Section 3 covers experimental setup and the method, Section 4 describes and discusses the results, and Section 5 offers conclusions.

## 2. Data and Data Preprocessing

### 2.1. Description of Data

The dataset, originally spanning a full calendar year from 1 March 2023 00:00:00 to 1 March 2024 00:00:00, was reduced to approximately ten and a half months to ensure data integrity and suitability for time series analysis covering the period from 31 March 2023 11:00:00 to 27 February 2024 18:00:00. This reduction was necessitated by the removal of extended gaps where interpolation was deemed infeasible due to the highly unsystematic and irregular nature of the time series.

The power plant operates across distinct operational regimes —load-following, startups, and shutdowns—with transient phases occurring irregularly in frequency and duration, complicating standard data preprocessing techniques. Given the temporal dependencies inherent in time series data, cross-validation was avoided, as recommended in literature for such unsystematic datasets [23], to prevent leakage of future information into training sets. Instead, a conventional 80-10-10 split (training, validation, test) was initially considered; however, to preserve the chronological integrity of startup and shutdown sequences, which are critical to capturing transient dynamics, the dataset was partitioned using only periods of stable operation as split points. This approach resulted in a revised distribution of 82% for the training set (378,991 samples), 7% for the validation set (32,086 samples), and 11% for the test set (52,261 samples), with the test set deliberately positioned at the end to reflect the standard practice of evaluating time series models on the most recent data.

Each subset includes both steady-state and transitional conditions. Based on batch counts, approximately 67% of the training set represents active turbine operation, and 33% corresponds to shutdown periods. The validation set contains 38% shutdown batches, and the test set 42%, ensuring representative coverage of transient behaviors. The validation and test sets intentionally contain a higher proportion of shutdown sequences. This overrepresentation reflects the natural variability in shutdown duration—some sequences are significantly longer than others—and was necessary to ensure that longer shutdowns were included. This design choice is critical, as our experiments showed that the model had far greater difficulty learning to predict shutdown conditions than startups and steady-state operation. By emphasizing these challenging periods in the evaluation subsets, we enabled a more rigorous assessment of the model’s generalization capacity under transient conditions.

The study employed a suite of sensors positioned around the medium-pressure turbine, with their specifications detailed in Table 3. These sensors provide critical data for real-time control, enabling precise tracking of the turbine’s behavior and its interaction with the broader electro-energy system. The intermediate-pressure (IP) parameters characterize the steam at the inlet of the IP turbine, while the low-pressure (LP) parameters describe the steam conditions at the IP turbine exit. The predicted temperature was available from two separate measurements, denoted as $t_{{\mathrm{casing}}_{1}}$ and $t_{{\mathrm{casing}}_{2}}$.

The measurement paths and control signals utilized in this study fall into two categories: physical measurements from the turbine and generator, and external signals issued by the central controller. Table 3 primarily contains the former, while the final two entries—set points for IP steam temperature ( °C) and electrical power output (MW)—represent the latter. Additionally, as direct measurement of the secondary steam flow through the medium-pressure turbine was not available, the explanatory variable $flow_{IP_{sec-steam}}$, included in Table 3, does not correspond to a direct sensor reading but is instead a value calculated based on multiple other sensors that were deliberately omitted from the table. These include the live steam flow sensor $flow_{live-steam}$, secondary steam flow sensors $flow_{10}$ and $flow_{20}$ typically associated with reheater or extraction line flows, as well as additional monitoring points such as $flow_{XW3}$, $flow_{XW4}$ (reheaters), and flow (steam header inlet). All physical thermocouples used were type K, class 1, which translates into an accuracy of +/−1.5 °C in the measurement range from −40 °C to 1000 °C.

### 2.2. Data Preprocessing

**Data Cleaning:** Raw data contained duplicated and irregular timestamps, non-numeric entries, and numerous missing values. Duplicate sensor measurements were averaged into a single signal. Signals with high missing rates were removed. The index was deduplicated and reindexed to ensure strictly increasing 1-min intervals.

**Interpolation:** Linear interpolation was then applied to all numeric columns. More complex methods such as PCHIP were considered but ultimately discarded due to their tendency to introduce unrealistic oscillations near flat regions.

**Feature Engineering:** Time-lagged features were generated for critical temperature signals. Additionally, smoothing was applied to selected features using both simple moving averages and exponentially weighted means. These smoothed signals were then log-transformed and differenced to extract the dynamic cooling coefficient k from Newton’s law of cooling [24]:

(1) $$T\left(t\right)=T_{\mathrm{ambient}}+\left(T_{0}-T_{\mathrm{ambient}}\right)\exp\left(-kt\right).$$

Strong smoothing was crucial for eliminating oscillations when the temperature approached ambient values, enabling tracking of how the cooling rate k evolved over time.

**RPM Signal Processing:** Shaft acceleration was estimated by differencing the RPM signal. Variations smaller than ±50 RPM were treated as zero, reflecting standard engineering practice of maintaining low-speed rotation during downtime to prevent mechanical stress [11]. The processed signal was discretized into 10-RPM bins.

**State Detection:** Operational states were labeled using domain-specific heuristics. Startup was defined as an RPM rise crossing the 50-RPM threshold following at least two hours of inactivity. This event was marked using a binary variable startup_180, which was also temporally extended to indicate a broader startup window. Shutdown events were identified using a binary flag shutdown, derived from smoothed RPM and blade temperature measurements, with a threshold on temperature decline to reliably detect the onset of cooling. The duration of each shutdown period was tracked using an auxiliary variable shut_minutes.

**Feature Scaling:** MinMax or MaxAbs scaling was chosen to normalize the input features, depending on the modeling setup. MaxAbs scaling preserves zero, which is critical for variables that zero out during shutdowns, ensuring this information is not lost. This choice was made because certain input columns—particularly those based on differences between time steps—exhibited very large dynamic ranges after standardization (zero mean, unit variance).

**Figure 3:**
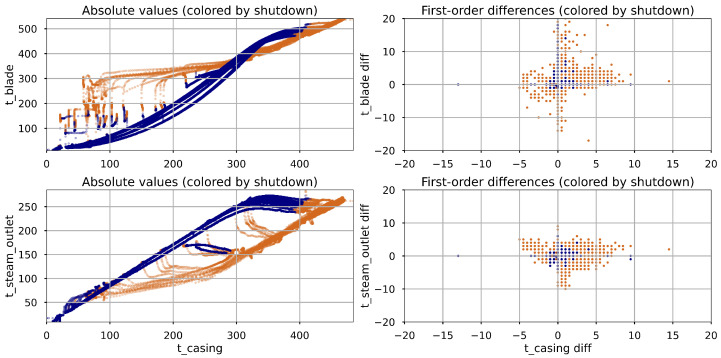
Scatter plots illustrating the relationship between turbine casing temperature and its two most strongly correlated variables. Data points from the shutdown phase are shown in navy, while those from the active phase are shown in brown.

**Correlation Analysis:** A correlation analysis was performed to examine the relationships between input features and turbine casing temperature ($t_{\mathrm{casing}}$), based on both absolute values and first-order differences. Results are presented in Table 4. For absolute values, $t_{\mathrm{blade}}$ and $t_{\mathrm{steam}\_\mathrm{outlet}}$ show very strong correlations with $t_{\mathrm{casing}}$ across all data segments, including during shutdowns (0.975 and 0.995 respectively), indicating that steady-state temperatures closely mirror casing temperature due to thermal inertia. In contrast, the correlations of IP and LP steam pressure, as well as RPM, drop substantially during shutdown (e.g., 0.149 and 0.263 for pressures, 0.472 for RPM), reflecting the fact that these signals remain nearly constant during shutdown, while temperatures continue to change, highlighting the dominance of temperature-driven dynamics in cooling phases. For first-order differences, all correlations are significantly lower than in the absolute case. The observed drop in correlation points to the likelihood of nonlinear relationships between $t_{\mathrm{casing}}$ and its predictors, especially under transient conditions. Scatter plots of $t_{\mathrm{casing}}$ versus $t_{\mathrm{blade}}$ and $t_{\mathrm{steam}\_\mathrm{outlet}}$ during shutdown (Figure 3) show curved trajectories, supporting the presence of nonlinear behavior due to thermal inertia. Moreover, shutdown-phase correlations fall drastically compared to active operation (e.g., 0.317 vs. 0.028 for $t_{\mathrm{blade}}$). These patterns reinforce the temperature-driven nature of casing behavior during shutdown and highlight the need for regime-specific models.

## 3. Methods

### 3.1. Dual Model Configuration

Building on the hysteresis identified in the correlation analysis (Section 2.2), where thermal inertia causes distinct relationships between variables during heating and cooling, we adopted a dual-model architecture to model these transient states separately. Two deep learning models were developed: $m_{\mathrm{shutdown}}$, trained on shutdown sequences, and $m_{\mathrm{active}}$, trained on all other sequences. Startup and load-following sequences were combined into $m_{\mathrm{active}}$ to maintain simplicity.

### 3.2. Temporal Sequence Preparation

The time series data were segmented into fixed-length input sequences using a sliding window approach. Each input sequence $X=[x_{1},\;x_{2},\;\ldots ,\;x_{L}]$ consists of *L* consecutive time steps, where each $x_{i}\in R^{F}$ represents a feature vector at the *i*-th time step (with *F* being the number of features). The corresponding prediction target—the turbine casing temperature $\left(t_{\mathrm{casing}}\right)$—was set as the value at the next time step L+1, forming input-output pairs $\left(X_{i},\;y_{i+L}\right)$, where $X_{i}$ is the input sequence of length *L* and $y_{i+L}$ is the scalar target value to be predicted.

Each sequence was assigned to either the $m_{\mathrm{shutdown}}$ or $m_{\mathrm{active}}$ model, depending on the value of the shutdown indicator within the window. If any time step in the sequence was marked as part of the shutdown phase, the entire sequence was labeled accordingly. The training set was shuffled to reduce temporal bias, while the validation and test sets preserved chronological order. Mini-batches were then constructed to improve computational efficiency during model training.

### 3.3. Neural Network Architecture

The core architecture for both $m_{\mathrm{shutdown}}$ and $m_{\mathrm{active}}$ models comprises a bidirectional Gated Recurrent Unit (GRU) layer, an Attention Mechanism layer, and a linear output layer, designed for temporal regression. While sharing this core architecture, the models were afforded the flexibility to vary in their GRU configurations, attention variants, and hyperparameters to optimize performance for their respective datasets.

#### 3.3.1. Bidirectional Recurrent Layer

Temporal feature extraction is facilitated by a bidirectional Gated Recurrent Unit (GRU) layer, configured with a symmetric unit allocation to capture both forward and backward temporal dependencies [25]. For an input sequence $X=[x_{1},\;x_{2},\;\ldots ,\;x_{L}]$, where $x_{i}\in R^{F}$, the GRU computes hidden states via a series of gated operations:

(2) $$z_{i}=\sigma \left(W_{z}x_{i}+U_{z}h_{i-1}+b_{z}\right),$$

(3) $$r_{i}=\sigma \left(W_{r}x_{i}+U_{r}h_{i-1}+b_{r}\right),$$

(4) $${\tilde{h}}_{i}=\tanh\left(W_{h}x_{i}+U_{h}\left(r_{i}⊙h_{i-1}\right)+b_{h}\right),$$

(5) $$h_{i}=\left(1-z_{i}\right)⊙h_{i-1}+z_{i}⊙{\tilde{h}}_{i},$$

where σ denotes the sigmoid function, ⊙ represents element-wise multiplication, and *W*, *U*, and *b* are trainable parameters governing the update ($z_{i}$), reset ($r_{i}$), and candidate state (${\tilde{h}}_{i}$). The bidirectional configuration concatenates forward and backward states, yielding a rich temporal representation.

#### 3.3.2. Attention Mechanism

To dynamically weight temporal contributions, an attention mechanism has been applied, prioritizing salient time steps within data sequences [22]. Three distinct variants were developed—treated as alternative configurations during hyperparameter tuning—each tailored to address different facets of temporal dynamics, particularly in the context of modeling dynamic systems.

**Feature attention (simple_AM)** computes attention weights based on a linear transformation of the input tensor $H\in R^{L\times D}$. Attention scores are calculated as follows:

(6) $$s_{i}=\tanh\left(HW+b\right),$$

where $H\in R^{L\times D}$ is the matrix whose rows are the hidden state vectors $h_{i}\in R^{D}$ (for *i* = 1, 2, …, *L*), $W\in R^{F\times 1}$ is a trainable weight matrix, and b∈R is a scalar bias broadcast across all time steps, yielding $s_{i}\in R^{L\times 1}$. *D* is the dimensionality of the hidden state vectors $h_{i}$, equal to twice the number of GRU units due to the bidirectional architecture.

(7) $$\alpha_{i}=\mathrm{softmax}\left(s_{i}\right)=\frac{\exp(s_{i})}{\sum_{j=1}^{T}\exp\left(s_{j}\right)},$$

producing attention weights $\alpha_{i}\in R^{L\times 1}$. The output is aggregated as follows:

(8) $$O=\sum_{i=1}^{T}\left(H_{i}⊙\alpha_{i}\right),$$

resulting in a fixed-dimensional vector $O\in R^{D}$. This variant prioritizes time steps based on learned feature significance, dynamically weighting moments where specific feature values are most predictive.

**Feature attention with short-term temporal dynamics (dir_AM)** extends the basic mechanism by incorporating directional dynamics through the inclusion of temporal gradients. For an input $H\in R^{L\times D}$, gradients are computed as follows:

(9) G=H[t+1,·]−H[t,·],t∈1,…,L−1

with padding to align dimensions. Attention scores combine contributions from values and gradients:

(10) $$s_{i}=\tanh\left(HW+GW_{\mathrm{grad}}+b\right),$$

where $W,W_{\mathrm{grad}}\in R^{F\times 1}$ are trainable weight matrices for values and gradients, respectively, and b∈R is a bias term. After softmax normalization, the weights $\alpha_{i}$ are applied to the input, producing the output:

(11) $$O=\sum_{i=1}^{T}\left(H_{i}⊙\alpha_{i}\right),$$

This variant focuses on time steps with rapid feature changes, such as sudden operational disruptions (e.g., a generator trip).

**Feature attention with short- and long-term temporal dynamics (long_dir_AM)** incorporates both short-term and long-term temporal gradients. Short-term gradients are computed as in dir_AM, while long-term gradients account for differences between points separated by up to *k* time steps:

(12) $$G_{\mathrm{long}}=H\left[t+k,\cdot \right]-H\left[t,\cdot \right],$$

with appropriate padding. Attention scores are calculated as follows:

(13) $$s_{i}=\tanh\left(HW+G_{\mathrm{short}}W_{grad_{short}}+G_{long}W_{grad_{long}}+b\right),$$

where $W,W_{grad_{short}},W_{grad_{long}}\in R^{F\times 1}$ are trainable weight matrices, and b∈R is a bias term. Following softmax normalization, the output is generated similarly to the other variants. This mechanism provides a broader temporal context, enhancing the model’s ability to anticipate trends.

#### 3.3.3. Output Layer

The terminal layer employs a single linear unit to map the attention-weighted representation to a scalar prediction:

(14) $$\hat{y}=W_{d}O+b_{d},$$

where $W_{d}\in R^{F\times 1}$ and $b_{d}\in R$ are optimized parameters and *O* is the output of the previous layer.

### 3.4. Model Optimization

To ensure optimal performance of the $m_{\mathrm{shutdown}}$ and $m_{\mathrm{active}}$ models, optimization was performed through feature selection and hyperparameter tuning, tailored to their stratified datasets (shutdown vs. active sequences, as described in Section 3.2).

A permutation-based method was applied to identify significant and non-redundant features for each model separately, mitigating neural network instability due to input variable correlations. The selected features, listed in Table 5, were combined into a single common set to simplify the model architecture. Subsequent permutation analysis confirmed that this common set did not degrade the performance of either $m_{\mathrm{shutdown}}$ or $m_{\mathrm{active}}$.

Hyperparameter tuning was conducted using the Hyperband algorithm, separately for each model, accounting for their distinct datasets. To determine the optimal sequence length, the tuning was performed twice for the model, evaluating sequence lengths (*L*) of 15 and 30 time steps. Different sequence lengths for the models were infeasible due to input data structure constraints, which required identical input shapes for $m_{\mathrm{shutdown}}$ and $m_{\mathrm{active}}$. Consequently, L=30 was chosen for both models.

The Hyperband search space encompassed architectural and training hyperparameters, as summarized in Table 6. Each tuning trial was limited to a maximum of 50 epochs, with early stopping and learning rate reduction callbacks applied to prevent overfitting and accelerate convergence. A maximum of 15 trials per model per *L* was conducted to balance exploration and computational efficiency.

The best hyperparameters for each model were selected based on the lowest value of the composite criterion for the chosen *L* (Section 3.5).

### 3.5. Comparison with Baseline Models

To evaluate the performance of the proposed method, it was compared against several established machine learning models: Random Forest Regressor (RFR), XGBoost, LSTM, and a variant of the proposed model trained on the entire dataset without splitting into shutdown and active regimes (Single Attentive Bi-GRU, SA Bi-GRU).

**RFR:** RFR uses variance reduction to assess feature importance, making it effective for capturing nonlinear relationships in time series data. The importance of a feature $X_{j}$ is computed as follows:

(15) $$\mathrm{Importance}\left(X_{j}\right)=\frac{1}{N_{T}}\sum_{T\in \mathrm{Trees}}\sum_{s\in \mathrm{Splits}(X_{j})}\Delta \mathrm{Var}\left(s\right),$$

where ΔVar(s) is the variance reduction at split *s*, and $N_{T}$ is the number of trees.

**XGBoost:** XGBoost optimizes a regularized objective function, balancing prediction error and model complexity, which is particularly suitable for handling non-stationary industrial data. Its objective at iteration (*t*) is

(16) $$L^{\left(t\right)}=\sum_{i=1}^{n}l\left(y_{i},\hat{y}i^{\left(t\right)}\right)+\sum {k=1}^{t}\Omega \left(f_{k}\right),$$

where $\Omega \left(f_{k}\right)=\gamma T+\frac{1}{2}\lambda {\left|\omega \right|}^{2}$ regularizes tree complexity.

**LSTM:** LSTM captures long-term temporal dependencies through its gating mechanisms, suitable for sequences like those in $m_{\mathrm{shutdown}}$. Its forget gate is defined as follows:

(17) $$f_{t}=\sigma \left(W_{f}\cdot \left[h_{t-1},x_{t}\right]+b_{f}\right).$$

**Single Attentive Bi-GRU:** This variant applies the proposed RNN architecture to the entire dataset without separating shutdown and active regimes, serving as a baseline to assess the benefit of the proposed split.

The specific hyperparameters for these models, listed in Table 7, were manually selected based on domain knowledge and preliminary experiments.

It is important to note that, unlike deep learning models, ensemble tree-based models such as Random Forest Regressor and XGBoost do not have a corresponding “dual” version for comparison. This limitation arises from the fact that ensemble tree-based models, classified as static machine learning (ML) models, operate on continuous time series data without inherently capturing temporal dynamics [2]. Removing segments corresponding to shutdown periods would create temporal discontinuities, thereby compromising the integrity of the input. In contrast, sequence-based deep models, which fall under dynamic ML models due to their ability to map temporal dependencies (e.g., through recurrent or convolutional architectures), offer the flexibility of constructing input sequences before any filtering is applied [2]. This design preserves the chronological structure of the data and avoids introducing gaps, enabling the effective implementation of a dual-model strategy.

To mitigate this limitation, potential workarounds for ensemble tree-based models could be explored. One approach involves imputing missing shutdown data with synthetic values to maintain continuity, though this risks introducing bias. Another option involves using strategies such as performance-based weighting or meta-learning [26]. However, applying ensembles to sequential data remains challenging due to the loss of temporal integrity [27].

### 3.6. Evaluation and Training

During the training phase, model performance was assessed based on the following metrics: Mean Absolute Error (MAE), the primary metric, measuring average prediction error:

(18) $$\mathrm{MAE}=\frac{1}{N}\sum_{i=1}^{N}\left|y_{i}-{\hat{y}}_{i}\right|,$$

where $y_{i}$ is the true value, ${\hat{y}}_{i}$ is the predicted value, and *N* is the number of samples. Mean Squared Error (MSE), used as the loss function to penalize larger errors:

(19) $$\mathrm{MSE}=\frac{1}{N}\sum_{i=1}^{N}{\left(y_{i}-{\hat{y}}_{i}\right)}^{2}.$$

The training paradigm employed RMSprop with momentum-augmented gradient descent, incorporating:

- **Early stopping**: Training halts if validation loss does not improve for a specified number of epochs. The model is restored from the best epoch.
- **Learning rate scheduling**: The learning rate $\eta_{t}$ at epoch *t* is reduced when validation loss plateaus:
  (20) $$\eta_{t}=\eta_{0}\cdot \gamma^{\left\lfloor t/p\right\rfloor },$$
  where $\eta_{0}$ is the initial rate, γ<1 is the decay factor, and *p* is the patience.
- **Dropout**: Deactivates a fraction $p_{\mathrm{drop}}$ of units during training to reduce overfitting:
  (21) $$h_{i}^{\prime }=h_{i}\cdot m_{i},\;m_{i}∼\mathrm{Bernoulli}\left(1-p_{\mathrm{drop}}\right),$$
  where $h_{i}$ is the unit output, and $m_{i}$ is a binary mask.
- **Momentum**: Stabilizes updates using a moving average of gradients:
  (22) $$v_{t}=\mu v_{t-1}+\left(1-\mu \right)\nabla \theta_{t},\;\theta_{t+1}=\theta_{t}-\eta v_{t},$$
  where $v_{t}$ is velocity, μ is the momentum coefficient, and η is the learning rate.

Experiments were repeated 10 times to mitigate randomness, and average performance metrics were calculated for statistical robustness. All experiments were implemented in KERAS with framework-default random weights initialization using Python 3.8 and Intel Core i5-6600 CPU (Intel, Santa Clara, CA, USA) and NVIDIA-RTX 3070 machine (NVIDIA, Santa Clara, CA, USA).

## 4. Results and Discussion

### 4.1. Model Performance Analysis

**Figure 4:**
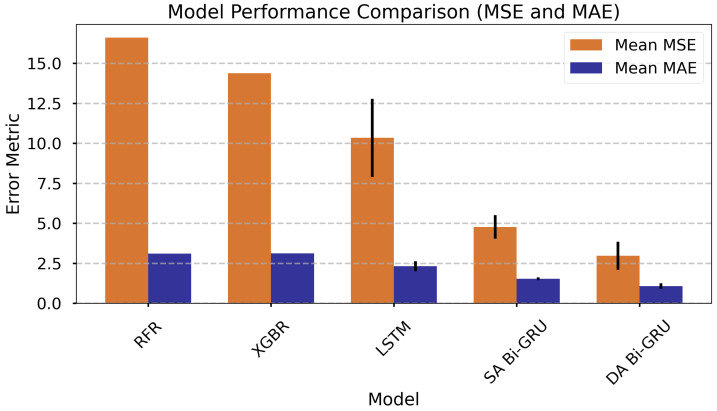
Comparison of model performance metrics, sorted by mean MSE (highest to lowest). *DA Bi-GRU* denotes Dual-Attention Bi-GRU; *SA Bi-GRU* denotes Single-Attention Bi-GRU.

The performance of the evaluated models is summarized in Table 7, which presents key metrics including *Mean Squared Error* (MSE), *Mean Absolute Error* (MAE), *Root Mean Squared Error* (RMSE), $R^{2}$, and *Mean Absolute Percentage Error* (MAPE). The models are sorted by mean MSE from highest (least accurate) to lowest (most accurate), as MSE is a critical indicator of predictive accuracy. The sorted results are also presented in a bar chart for visual comparison, as shown in Figure 4.

Although standard deviations are comprehensively reported in Table 8, they are omitted for RFR and XGBoost due to the deterministic nature of these ensemble models. Their performance remains highly stable across runs, in contrast to deep learning models, where stochastic elements—such as random initialization, dropout, and mini-batch sampling—introduce significant variability.

The RFR model exhibits the highest MSE of 16.61, indicating the least accurate predictions among the models. XGBoost follows with an MSE of 14.38, slightly better but still suboptimal. LSTM, with an MSE of 10.34, shows improved performance but has a notable standard deviation of 2.44, suggesting variability in its predictions. The *Single Attentive Bi-GRU* model further improves with an MSE of 4.77, while the *Dual Attentive Bi-GRU* model achieves the lowest MSE of 2.97, demonstrating superior predictive accuracy. The standard deviations of MSE and MAE for the *Dual Attentive Bi-GRU* (0.88) are significantly lower than those of the LSTM, indicating more consistent performance; however, they are slightly higher compared to the *Single Attentive Bi-GRU*.

Regarding MAE, the *Dual Attentive Bi-GRU* model again performs best, with a mean MAE of 1.07 compared to roughly 3.1 for both RFR and XGBoost. The MAPE metric follows a similar trend, with *Dual Attentive Bi-GRU* achieving the lowest value (0.39). All models report an $R^{2}$ close to 1.00, indicating an excellent fit to the data. The *Dual Attentive Bi-GRU* model’s consistent metrics across the board highlight its robustness for predictive tasks, making it the most suitable choice among the evaluated models.

**Figure 5:**
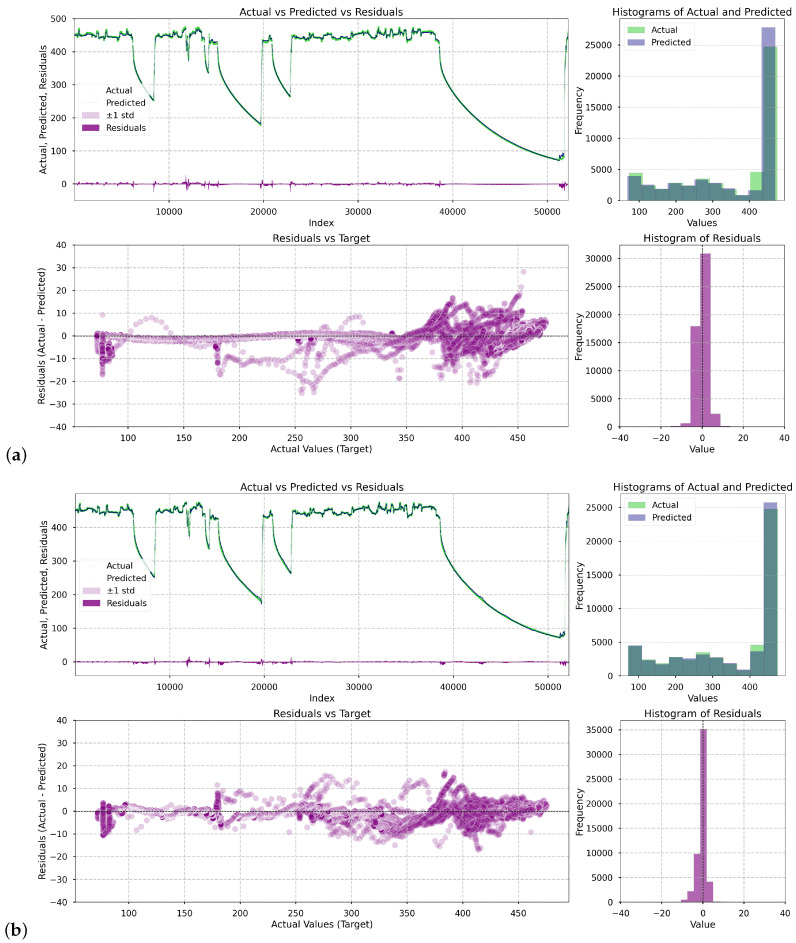

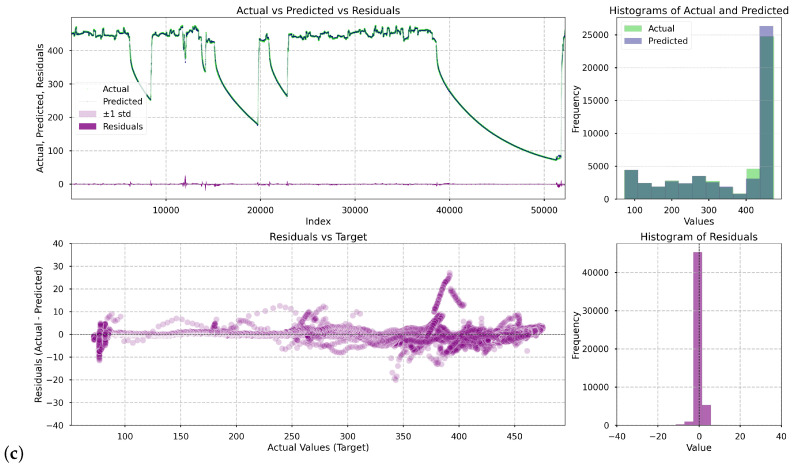
Prediction and residual analysis for the deep learning models: (**a**) LSTM, (**b**) Single Attentive Bi-GRU, (**c**) Dual Attentive Bi-GRU. The subplots include: (1) actual vs. predicted values over time, (2) residuals vs. predicted values, (3) overlaid histograms of actual and predicted distributions, and (4) histogram of residuals.

Figure 5 presents a series of visualizations illustrating how the deep learning models performed in the prediction task. For each of them, i.e., LSTM, Single Attentive Bi-GRU, Dual Attentive Bi-GRU, four plots were generated: (1) a comparison of actual (green) versus predicted (black) values of the casing temperature and versus residuals (purple) over time, (2) overlaid histograms showing the distribution of actual and predicted values, (3) a scatter plot showing the residuals as a function of the turbine casing temperature, and (4) a histogram of residuals.

As shown in the presented plots, each successive model demonstrates increasingly better predictive performance. The overall fit improves progressively, with residuals becoming smaller and more symmetrically distributed. The only regions where notable errors persist are narrow spikes in the residual plots. These occur specifically at the onset of turbine startups—short transitional periods when the system switches operational regimes. Nevertheless, errors in these regions are of limited practical concern. From an operational perspective, an increase in temperature during startup is expected and managed by the plant’s automation systems. Moreover, we attempted to enhance model performance by including the variable startup_180, which indicates that a turbine startup sequence is imminent (cf. Section 2.2 *State Detection*). However, this feature was consistently deemed uninformative during training. This is likely because the anticipated startup was already implicitly captured by the power setpoint variable ${\mathrm{SP}}_{\mathrm{power}}$, rendering startup_180 redundant.

The training process was configured with a maximum of 500 epochs, a value determined through extensive experimentation to provide a substantial margin for convergence. Early stopping was employed to prevent overfitting, halting training when no improvement in validation loss was observed. Consequently, the average number of epochs required for convergence was 132.40 for the shutdown dataset and 155.40 for the active dataset.

**Figure 6:**
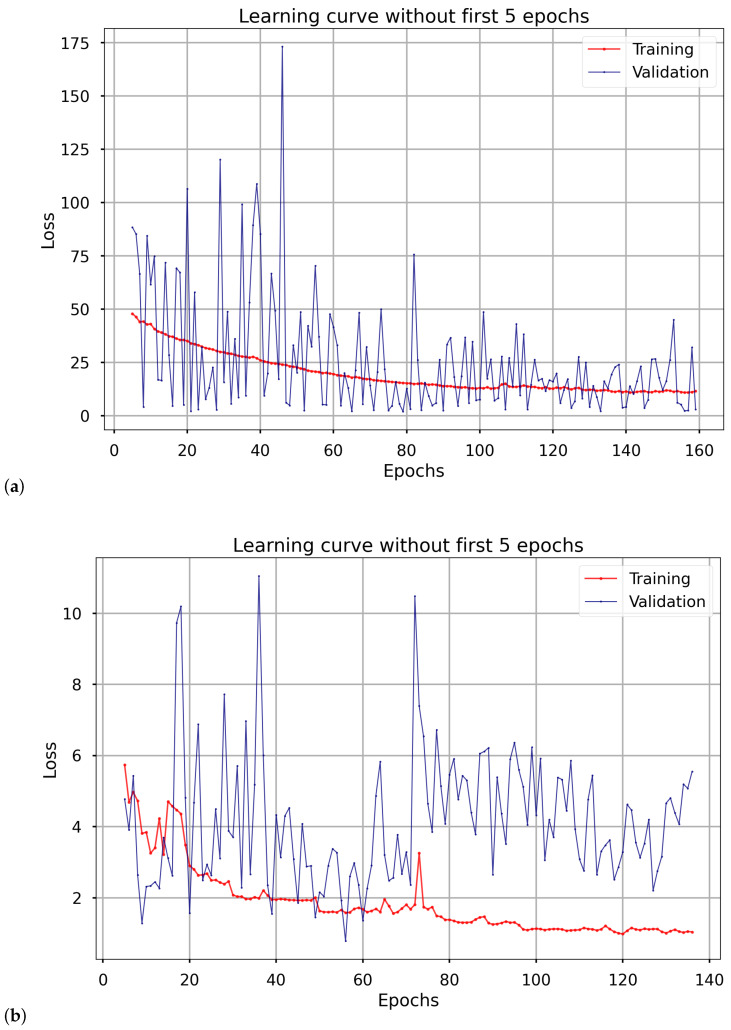
Prediction and residual analysis for the *Dual Attentive Bi-GRU* model. The subplots include (**a**) training curves for $m_{\mathrm{shutdown}}$ and (**b**) training curves for $m_{\mathrm{active}}$.

The learning curves depicted in Figure 6 illustrate the training and validation loss trends for the two models that Dual Attentive Bi-GRU is composed of $m_{\mathrm{shutdown}}$ (on the left side) and $m_{\mathrm{active}}$ (on the right side), with the initial five epochs excluded from the visualization. These are the learning curves from the second run and are fairly representative of other runs; in this run, the number of epochs for the respective models $m_{\mathrm{shutdown}}$ and $m_{\mathrm{active}}$ approximates their average epoch count, with a similar trend observed for the loss. The greater fluctuation in the validation loss curve for $m_{\mathrm{shutdown}}$ compared to $m_{\mathrm{active}}$ suggests that the shutdown data may exhibit more complex patterns. Higher variability in validation loss for the shutdown model could be due to the high proportion of inactive or low-information sequences. Although temperature profiles during shutdown are structurally similar, many 30-min windows occur during periods with minimal thermal activity, reducing the signal-to-noise ratio and potentially destabilizing the validation loss.

The observed difference in convergence epochs—132.40 for $m_{\mathrm{shutdown}}$ versus 155.40 for $m_{\mathrm{active}}$—may reflect several underlying factors. One contributing factor may be the higher initial learning rate used for $m_{\mathrm{shutdown}}$ (0.01 vs. 0.001 for $m_{\mathrm{active}}$), which can accelerate early-stage optimization and lead to convergence in fewer epochs. The higher proportion of shutdown sequences in the validation (38%) and test sets (42%) compared to the training set (33%, Section 2.1) likely enhances the validation performance of $m_{\mathrm{shutdown}}$, contributing to its faster convergence, though the heterogeneity of shutdown data (e.g., varying sequence lengths and cooling patterns) introduces variability in loss trends (Figure 6).

Given these multiple factors—data characteristics, model configuration, and training dynamics—a detailed analysis would benefit from additional experiments, such as comparing convergence with fixed hyperparameters or analyzing loss gradients, to isolate the dominant influence. Nevertheless, this difference suggests that $m_{\mathrm{shutdown}}$ may be more efficiently tuned to its specific regime, offering a potential area for further optimization in future studies.

### 4.2. Prediction Time Analysis

The prediction time of a model is a critical factor in evaluating its practical applicability, particularly for real-time or resource-constrained environments. Table 9 presents the mean prediction times, along with their standard deviations and other relevant metrics, for the evaluated models, sorted from the highest (least efficient) to the lowest (most efficient) mean prediction time.

**Figure 7:**
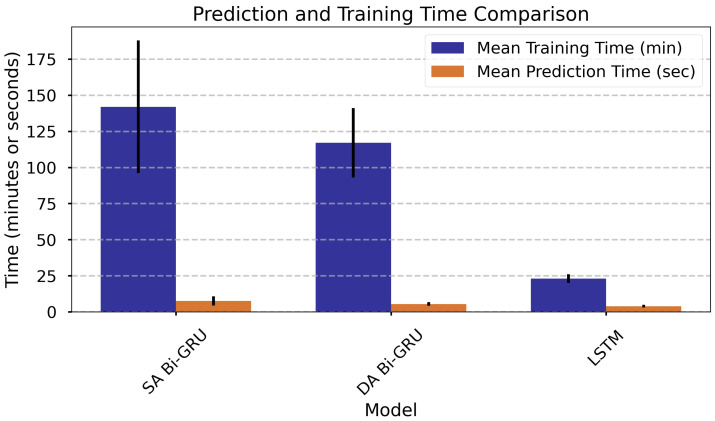
Comparison of model execution times and parameters, sorted by mean prediction time (highest to lowest). *DA Bi-GRU* denotes Dual-Attention Bi-GRU; *SA Bi-GRU* denotes Single-Attention Bi-GRU.

The Single-Attention Bi-GRU (SA Bi-GRU) model required the longest training time (142 min) and exhibited the highest standard deviation of 46 min. In contrast, the Dual-Attention Bi-GRU (DA Bi-GRU) trained faster (117 min) and with lower variation (24 min), despite having a nearly identical number of parameters. Also, the latter predicted more efficiently (5.5 s vs. 7.6 s). These differences likely stem from architectural and hyperparameter distinctions. The LSTM model was the fastest and most consistent, completing training in 23 min and inference in 3.9 s with a standard deviation of 0.9 s, owing to its simpler architecture and much smaller parameter set (1553). Overall, DA Bi-GRU offers an advantageous trade-off, combining richer architecture with competitive efficiency. These trends are also visualized in Figure 7.

### 4.3. Static Models Performance with Extended Feature Sets

To ensure a fair comparison between neural network models and ensemble tree-based models, both RFR and XGBoost were trained on all available explanatory variables, including lagged and smoothed features, increasing the feature set from 10 to 28 variables. Hyperparameters were adjusted accordingly to account for the enlarged feature space. Given the robustness of ensemble tree-based models to high-dimensional input spaces and their limited reliance on aggressive feature selection [28], this expanded feature approach allowed us to test whether incorporating additional temporal context or signal transformations could enhance predictive performance by capturing system dynamics using otherwise static models [26].

The results, however, showed only marginal improvement. For RFR, MSE decreased from 16.6 to 14.7, while for XGBoost, it dropped from 14.4 to 12.6. All the performance metrics remained nearly unchanged, suggesting that the additional features did not substantially improve model generalization and indicating potential redundancy in the extended feature set. These findings align with [26,27], who note that the temporal nature of data often poses challenges for tree-based ensembles, and that efforts to adapt them through transformations may not effectively enhance their predictive robustness. In contrast, the strong performance of the Dual Attentive Bi-GRU highlights the importance of explicitly modeling regime shifts.

### 4.4. Discussion and Future Work

While the proposed dual-model soft-sensing framework demonstrates high predictive accuracy and clear practical advantages, several limitations remain that warrant further study. First, the system’s performance has been validated on a single large-scale steam turbine unit. Although the architecture is conceptually generalizable, further testing on plants of different capacities, designs, and control strategies is required to confirm its scalability and robustness under broader industrial conditions. Second, like any data-driven system, the models depend on the availability and quality of sensor data. Unexpected sensor drift, missing signals, or unrepresented operating states may degrade performance if not detected early. Integrating automated drift detection, adaptive retraining, and hybrid physical–data-driven models could increase resilience and reduce maintenance demands. Third, real-time deployment introduces constraints related to computational efficiency and power consumption, especially if the models are embedded directly in control hardware or edge devices. Future work could explore model compression, quantization, and lightweight attention mechanisms to maintain inference speed while reducing resource usage. Additionally, coupling such models with uncertainty quantification and physics-informed learning would not only improve interpretability but also strengthen trust and safety in production environments.

## 5. Conclusions

The proposed dual-model soft-sensing framework provides an effective and operationally viable solution for estimating steam turbine casing temperatures under diverse regimes. By explicitly distinguishing between shutdown and active operating states, the method captures the thermal hysteresis that conventional single-model approaches often overlook. This regime-aware structure enhances predictive accuracy while retaining physical interpretability, offering a pathway toward reliable, real-time temperature monitoring in large-scale power plants. Beyond the numerical improvements presented in Section 4, the approach holds several promising applications. It may be implemented as a virtual sensor to replace or supplement damaged thermocouples, as a diagnostic tool for detecting abnormal thermal gradients during startup or load transients, or as a module within a digital twin for predicting material fatigue and optimizing operational scheduling. Its computational efficiency enables on-site integration with existing DCS/SCADA systems, making it suitable for online advisory or supervisory control functions.

However, several limitations merit attention. The current validation was conducted on a single 370 MW unit, and further verification across turbines of varying design and capacity is necessary to confirm scalability. The model’s dependence on data quality means that long-term reliability will require mechanisms for continuous drift detection, retraining, and data quality assurance. While the framework demonstrates robustness under typical transient conditions, its extrapolation beyond the training domain—such as during emergency trips or sensor malfunctions—remains constrained. Integrating uncertainty quantification and adaptive recalibration routines would mitigate these risks. Future research should also address the computational and energy efficiency of the system when deployed at scale. Investigating model compression, attention-pruning, and hybrid physics-informed architectures could balance inference cost with predictive accuracy. Furthermore, extending the framework toward multi-component modeling—linking turbine dynamics with boiler, condenser, or feedwater systems—could yield more comprehensive digital representations of the thermodynamic cycle. In essence, the dual-model concept presented here is not only a soft-sensing solution but also a general methodology for embedding physical reasoning into data-driven models. Its successful deployment may mark a step toward intelligent, self-adaptive monitoring systems capable of supporting safer and more efficient operation of future energy infrastructures.

## Figures and Tables

**Table 1:** Comparison of TG4 turbine startups by type for the years 2023 and 2024 [10].

Startup Type	2023	2024
Cold Start	15	21
Warm Start	8	15
Hot Start	2	3
**Total Startups**	**25**	**39**

**Table 2:** Relationship between IP steam temperature and power output under turbine control [10].

Power [MW]	IP Steam Temperature [°C]
100	416
150	455
200	482
250	502
300	520
370	535

**Table 3:** List of variables used in the soft sensor model.

Measurement	Variable	Min	Max	Resolution	Unit	Type
Electrical Power	power	0	400	1 min	MW	Sensor
Turbine Rotation	RPM	0	3500	1 min	$\min^{-1}$	Sensor
Inner Casing Temperature (1)	$t_{{\mathrm{casing}}_{1}}$	0	600	1 min	°C	Sensor
Inner Casing Temperature (2)	$t_{{\mathrm{casing}}_{2}}$	0	600	1 min	°C	Sensor
Blade Temperature	$t_{\mathrm{blade}}$	0	600	1 min	°C	Sensor
IP Steam Pressure	$p_{\mathrm{steam}\_\mathrm{inlet}}$	0	6	1 min	MPa	Sensor
IP Steam Temperature	$t_{\mathrm{steam}\_\mathrm{inlet}}$	0	300	1 min	°C	Sensor
LP Steam Pressure (A)	$p_{\mathrm{steam}\_\mathrm{outlet}\_A}$	0	6	1 min	MPa	Sensor
LP Steam Pressure (B)	$p_{\mathrm{steam}\_\mathrm{outlet}\_B}$	0	6	1 min	MPa	Sensor
LP Steam Temperature	$t_{\mathrm{steam}\_\mathrm{outlet}}$	0	300	1 min	°C	Sensor
Live Steam Flow	${\mathrm{flow}}_{\mathrm{live}-\mathrm{steam}}$	0	1600	1 min	t/h	Sensor
Secondary Steam Flow 10	${\mathrm{flow}}_{10}$	−0.45	45.45	1 min	t/h	Sensor
Secondary Steam Flow 20	${\mathrm{flow}}_{20}$	−0.5	50.5	1 min	t/h	Sensor
Auxiliary Flow XW3	${\mathrm{flow}}_{\mathrm{XW}3}$	0	60	1 min	t/h	Sensor
Auxiliary Flow XW4	${\mathrm{flow}}_{\mathrm{XW}4}$	0	60	1 min	t/h	Sensor
Steam Header Flow	flow	0	120	1 min	t/h	Sensor
Calculated IP Steam Flow	${\mathrm{flow}}_{\mathrm{steam}\_\mathrm{inlet}}$	–	–	1 min	t/h	Calculated
IP Steam Temp Set Point	${\mathrm{SP}}_{\mathrm{steam}\_\mathrm{inlet}}$	350	600	1 min	°C	Setpoint
Electrical Power Set Point	${\mathrm{SP}}_{\mathrm{power}}$	0	400	1 min	MW	Setpoint

**Table 4:** Pearson Correlation Coefficients with Turbine Casing Temperature ($t_{\mathrm{casing}}$).

Feature	Absolute Values			First Differences		
	**All**	**Active**	**Shutdown**	**All**	**Active**	**Shutdown**
$t_{\mathrm{blade}}$	0.991	0.984	0.975	0.263	0.317	0.028
$t_{\mathrm{steam}\_\mathrm{outlet}}$	0.961	0.986	0.995	0.189	0.223	0.017
$p_{\mathrm{steam}\_\mathrm{intlet}}$	0.829	0.647	0.149	0.149	0.168	0.019
$p_{\mathrm{steam}\_\mathrm{outlet}\_A}$	0.810	0.646	0.263	0.170	0.188	0.015
$t_{\mathrm{power}}$	0.832	0.727	NaN	0.176	0.193	NaN
$t_{\mathrm{RPM}}$	0.855	0.816	0.472	0.199	0.221	0.016

**Table 5:** Features selected for the model.

Model	Features
$m_{\mathrm{active}}$	$t_{\mathrm{blade}},\;t_{\mathrm{steam}\_\mathrm{outlet}},\;power,\;SP_{power},\;RPM$, shutdown, startup_180
$m_{\mathrm{shutdown}}$	$t_{\mathrm{blade}},\;t_{\mathrm{blade}}^{5\mathrm{smth}},\;t_{\mathrm{steam}\_\mathrm{outlet}},\;t_{\mathrm{steam}\_\mathrm{outlet}}^{5\mathrm{smth}}$, shutdown, shut_minutes

**Table 6:** Final hyperparameter configurations selected via Hyperband (L=30).

Hp	Description	$m_{\mathrm{Shutdown}}$	$m_{\mathrm{Active}}$
${\mathrm{variant}}_{\mathrm{gru}}$	Number of GRU layers	1xGRU	1xGRU
${\mathrm{variant}}_{\mathrm{nodes}}$	Number of GRU units	64	32
${\mathrm{variant}}_{\mathrm{am}}$	Type of Attention Mechanism	dir_AM	simple_AM
$\eta_{0}$	Initial learning rate	0.01	0.001
μ	Momentum coefficient	0.8	0.8
$p_{\mathrm{drop}}$	Dropout rate	0.0	0.0
regl2	L2 regularization strength	0.0	0.0
${\mathrm{patience}}_{\mathrm{lr}}$	LR reduction patience (epochs)	20	10
${\mathrm{patience}}_{\mathrm{es}}$	Early stopping patience (epochs)	80	80
$\min_{\delta }$	Minimum loss improvement threshold	0.1	0.01

**Table 7:** Hyperparameters for baseline models.

Model	Hyperparameters
RFR	Number of trees: 100, Min samples per leaf: 1, Out-of-bag score enabled, Max depth: 15, Max features: 0.5
XGBoost	Learning rate: 0.01, Number of trees: 100, Max depth: 15, Subsample: 0.5, Column sample by tree: 0.5, L1 regularization: 10, L2 regularization: 10
LSTM (L=30)	Number of nodes: 16, Initial learning rate: 0.001, Momentum: 0.5, Dropout: 0.2, L2 regularization: 0.0, Patience for LR scheduler: 10, Patience for early stopping: 20, Min delta: 0.01
SA Bi-GRU (L=30)	Architecture: 1xGRU + simple_AM, Number of nodes: 64, Initial learning rate: 0.01, Momentum: 0.8, Dropout: 0.4, L2 regularization: 0.0, Patience for LR scheduler: 80, Patience for early stopping: 80, Min delta: 0.01

**Table 8:** Comparison of model performance metrics, sorted by mean MSE (highest to lowest). *DA Bi-GRU* denotes Dual-Attention Bi-GRU; *SA Bi-GRU* denotes Single-Attention Bi-GRU.

Model	MSE	MSE Std	MAE	MAE Std	RMSE	RMSE	$R^{2}$	MAPE	MAPE Std
RFR	16.61	—	3.10	—	4.08	—	0.9990	0.89	—
XGBoost	14.38	—	3.12	—	3.79	—	0.9992	0.90	—
LSTM	10.34	2.44	2.31	0.31	3.19	0.39	0.9994	0.82	0.12
SA Bi-GRU	4.77	0.74	1.53	0.09	2.18	0.17	0.9997	0.57	0.06
DA Bi-GRU	2.97	0.88	1.07	0.17	1.71	0.25	0.9998	0.39	0.06

**Table 9:** Comparison of model execution times and parameters, sorted by mean prediction time (highest to lowest). *DA Bi-GRU* denotes Dual-Attention Bi-GRU; *SA Bi-GRU* denotes Single-Attention Bi-GRU.

Model	Train Time (h)	Train Std (h)	Predict Time (s)	Predict Std (s)	Number of Params
SA Bi-GRU	2.36	0.76	7.59	3.20	28,290
DA Bi-GRU	1.95	0.41	5.45	1.23	28,418
LSTM	0.39	0.04	3.93	0.90	1553

## Data Availability

The original contributions presented in this study are included in the article. Further inquiries can be directed to the corresponding author.
